# Alterations in the Crystallization Pattern of Tear Fluid Induced by Increases in the Body Mass Index

**DOI:** 10.3390/life16020210

**Published:** 2026-01-27

**Authors:** Cosmin Victor Ganea, Corina Georgiana Bogdanici, Nicoleta Anton, Calina Anda Sandu, Ioana Madalina Bilha, Anisia Iuliana Alexa, Vlad Constantin Donica, Irina Andreea Pavel, Roxana Elena Ciuntu, Camelia Margareta Bogdanici

**Affiliations:** 1 Doctoral School, Grigore T. Popa University of Medicine and Pharmacy, University Street No. 16, 700115 Iasi, Romania; cosmin-victor.ganea@umfiasi.ro (C.V.G.); calina-anda.sandu@umfiasi.ro (C.A.S.); madalinabilha@gmail.com (I.M.B.); 2 Department of Ophthalmology, Faculty of Medicine, Grigore T. Popa University of Medicine and Pharmacy, University Street No. 16, 700115 Iasi, Romania; anton.nicoleta1@umfiasi.ro (N.A.); anisia-iuliana.alexa@umfiasi.ro (A.I.A.); vlad-constantin.donica@umfiasi.ro (V.C.D.); andreea.niagu@umfiasi.ro (I.A.P.); roxana-elena.ciuntu@umfiasi.ro (R.E.C.); camelia.bogdanici@umfiasi.ro (C.M.B.)

**Keywords:** body mass index, tear ferning test, dry eye, diagnostic patterning tests, obesity

## Abstract

(1) Purpose: The study investigated the correlation between variations in body mass index (BMI) and tear crystallization class according to the Masmali classification. Moreover, it examined the potential diagnostic value of a patterning test within the population affected by obesity. (2) Methods: A total of 61 patients were investigated, with ages ranging from 25 to 72 years (median age [interquartile range] = 39.0 [26] years). BMI values ranged from 19.1 to 47.5 kg/m^2^, with a median BMI (interquartile range) of 29.3 (12.1) kg/m^2^. (3) Results: The Kruskal–Wallis test was used to assess differences in BMI across the Masmali classes and revealed statistically significant disparities between at least two groups (*p* = 0.024). The rank η^2^ value of 0.094 shows a small-to-moderate effect size, suggesting that approximately 9% of the variance in rank distributions is explained by the Masmali classification. The post hoc Dunn test with Bonferroni and Holm corrections showed that patients classified as Masmali grade 2 exhibited a significantly higher BMI compared to those in grade 0 (*p* = 0.009), whereas no statistically significant differences were identified between grades 0 and 1 or between grades 1 and 2. The analysis of variance (ANOVA) revealed a statistically significant difference among Masmali Classes 0, 1, and 2 with respect to the number of branching structures in dried tear samples analyzed at a brightness threshold of 220. The effect size (η^2^ = 0.263) shows that approximately 26% of the variability in branching number can be attributed to the severity of tear film dysfunction as defined by the Masmali classification. Accordingly, as the Masmali score increases, the number of branches decreases significantly, particularly among patients with elevated BMI. (4) Conclusion: The findings suggest that when classified according to the Masmali scale the dry eye syndrome exhibits a distinct crystallization pattern in patients with excess body weight. Specifically, higher BMI values are associated with a marked decrease in the number of fern-like branches identified in the tear ferning test.

## 1. Introduction

Dietary changes and hydration status are reflected both systemically and at the level of the tear film [1,2]. Weight gain affects the entire body. Locally, at the ocular surface, a state of inflammation develops, predisposing patients to the onset of obesity-associated dry eye syndrome [3].

Diagnostic patterning tests (DPT) enable the identification of pathological conditions based on the spatial arrangement of fern-like structures formed during the crystallization of salts present in the analyzed biological fluid [4]. These tests have been applied to the study of multiple body fluids, including blood, plasma, tears, cerebrospinal fluid, saliva, sweat, urine, and cervical mucus. Their diagnostic value has been demonstrated across several medical fields, such as ophthalmology, gynecology, endocrinology, diabetology, and oncology [5,6,7].

In 2015 Masmali et al. introduced a revised five-tier classification system for the tear ferning test. In Class 0, the ferning phenomenon is fully developed. In Class 1, small empty spaces occur between crystallization patterns. In Class 2, the branches become thicker and the interbranch spaces widen. Class 3 shows an absence of branching, with only crystal aggregates present. In Class 4, the ferning phenomenon is completely absent [8].

The tear ferning test may serve as a valuable clinical tool for evaluating tear film quality. Importantly, it is not influenced by ocular surface stimulation and shows a high degree of inter-ocular consistency in crystallization patterns [9].

Previous studies have shown a connection between higher BMI and tear film alterations measured with the tear ferning test. However, most of that work relied on qualitative grading systems, which are inherently subjective and reduce reproducibility. For this reason, the central aim of the present study was not to reconfirm the BMI–dry eye relationship, but rather to introduce and implement a quantitative, image-based method for analyzing tear ferning. Using ImageJ2 (version 1.53), we objectively measured the number of ferning branches under standardized conditions and assessed how these values corresponded to tear film dysfunction severity, as defined by the Masmali scale.

## 2. Materials and Methods

The study design and protocol were performed according to the tenets of the Declaration of Helsinki for research involving human subjects and approved by the Ethics Committee of “Grigore T. Popa” University of Medicine and Pharmacy Iasi, Romania (No. 663/approval date on 28 October 2025). Written informed consent was obtained prior to patient evaluation.

### 2.1. Subjects

A total of 61 patients aged between 25 and 72 years were examined (median age [interquartile range] = 39.0 [26] years). BMI values ranged from 19.1 to 47.5 kg/m^2^, with a median BMI (interquartile range) of 29.3 (12.1) kg/m^2^.

### 2.2. Inclusion Criteria

We selected patients presenting for their first ophthalmological examination, aged between 25 and 72 years, who provided informed consent and showed willingness to comply with the pre-test instructions (abstaining from the use of cosmetic products on the day of tear collection, and observing an overnight fast from both food and liquids prior to body composition analysis).

### 2.3. Exclusion Criteria

Patients who had used contact lenses within the preceding week, those undergoing treatment with artificial tears, or patients with a history of ocular surgery were excluded from the study. Likewise, patients with systemic diseases known to affect the ocular surface, such as Sjögren’s syndrome or sarcoidosis, as well as those who were pregnant, smokers or previously diagnosed with dry eye syndrome or diabetes, were not enrolled.

Smokers, patients with diabetes mellitus, and individuals previously diagnosed with dry eye disease were excluded to minimize potential confounding, as each of these conditions is independently linked to tear film instability, ocular surface inflammation, and altered tear-ferning patterns. This strategy allowed us to more clearly isolate the relationship between BMI and tear crystallization characteristics.

### 2.4. BMI

Patients were classified according to BMI using the following criteria: underweight (15–19.9 kg/m^2^), normal weight (20–24.9 kg/m^2^), overweight (25–29.9 kg/m^2^), and obesity (≥30 kg/m^2^) [3]. BMI measurements were performed using a Tanita BC-545N Digital scale (Tokyo, Japan) and a Seca 206 stadiometer (Seca GmbH & Co. KG, Hamburg, Germany).

### 2.5. Tear Ferning Test

The tear ferning test (TFT) involves dry tear samples followed by microscopic examination. A standardized volume of 3 μL was collected using a 10 μL micropipette tip (DLAB Scientific Co., Ltd., Beijing, China), through vacuum generated by the piston returning to its initial position. Tears were obtained from the lateral canthus to avoid the risk of corneal injury. The desiccation process required 10 min under controlled environmental conditions (24 °C, 46% humidity). Samples were examined using a Levenhuk MED D45T Digital Trinocular phase-contrast microscope (Levenhuk, Tampa, FL, USA). Images were captured with the primary lens of an iPhone 15 Pro Max connected to the microscope via an ocular adapter. Phase-contrast microscopy is recommended for the evaluation of dried tear samples, as it enhances contrast and improves interpretative sensitivity [8].

### 2.6. Classification of the Tear Samples

We consider the updated Masmali classification to be superior to the traditional Rolando system in its discriminatory capacity for tear-drying patterns. For this reason, patient tear samples were examined using the Tear Ferning Test and subsequently categorized into Masmali classes 0, 1, and 2; it is noteworthy that no subjects exhibited Masmali classes 3 or 4. This study investigated whether the Tear Ferning Test could serve as a body-composition assessment tool in patients with altered weight status.

### 2.7. Statistical Analysis

Data were processed and analyzed using the statistical software JASP Team (2024), JASP (Version 0.19.2) [Computer software], with the significance threshold set at 95%. A *p*-value < 0.05 was considered statistically significant. Data distribution was evaluated using the Shapiro–Wilk test.

During the statistical analysis, two separate one-way ANOVA tests were performed—one for each dependent variable (BMI and the number of tear-fern branches quantified at Brightness 220)—using the Masmali classification as the common grouping factor. Because the variables examined represent distinct phenomena, no multiplicity correction was required.

The authors used OpenAI (ChatGPT 5.2) to assist in the interpretation of statistical results by comparing AI-generated interpretations with the authors’ own analysis. The final interpretation, conclusions and responsibility for the content rest entirely with the authors.

## 3. Results

**Figure 1:**
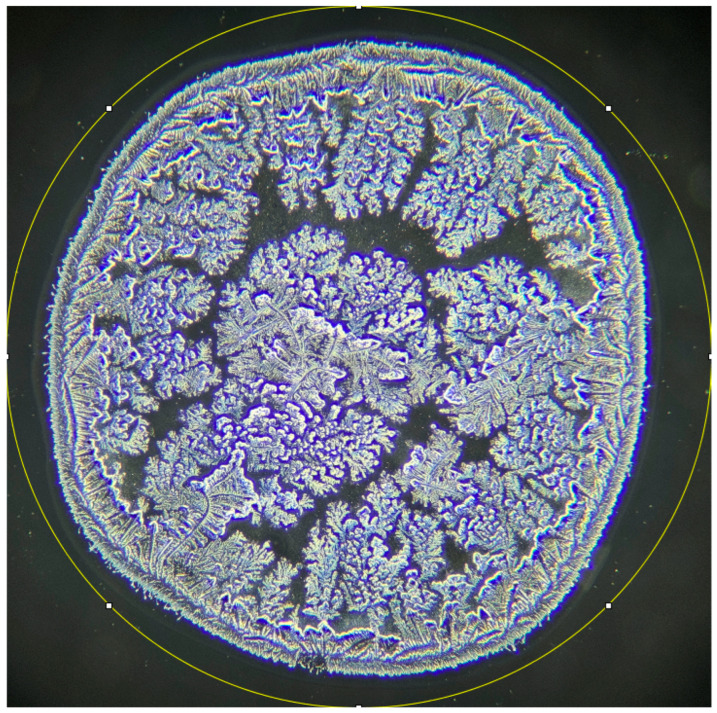
Selection of the region of interest for subsequent analysis. Phase-contrast microscopic appearance of the dried tear sample.

The following results were obtained through the photographic documentation of dried tears within the framework of the Tear Ferning Test. To enhance the rigor of the research and, implicitly, the reproducibility of the investigation, Masmali classes 0, 1, and 2 were analyzed in patients with altered body weight according to the brightness intensity of the processed images in ImageJ2. Initially, the area of interest was selected for analysis (Figure 1).

**Figure 2:**
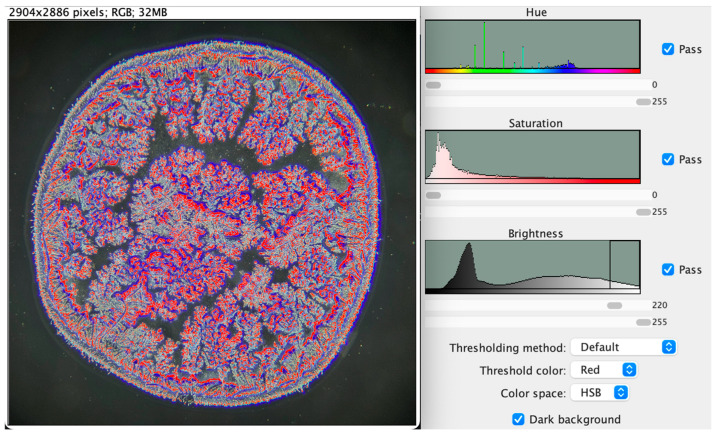
Brightness-based adjustment used to isolate the tear crystallization pattern.

**Figure 3:**
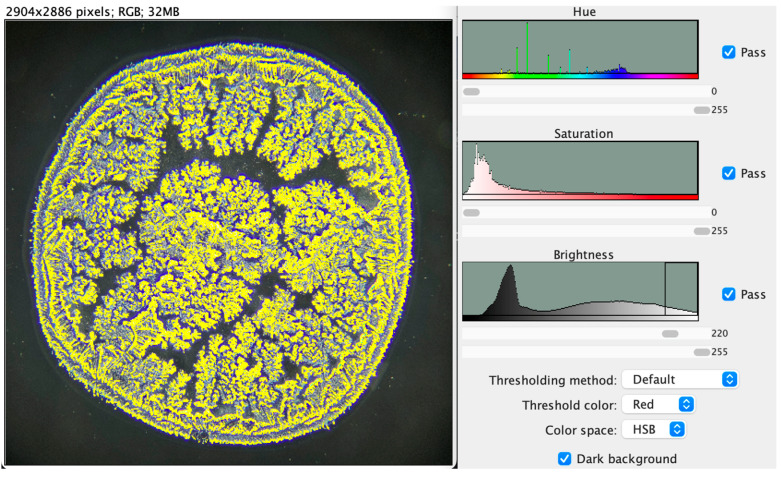
Brightness-threshold selection of the crystallization pattern within the tear ferning pattern.

The images were standardized by adjusting and selecting a brightness threshold of 220 (Figure 2 and Figure 3), which represents the lowest parameter capable of reliably identifying crystallization patterns. The number of branches was subsequently calculated at multiple brightness thresholds, decreasing in increments of 5 from 220 to 175. However, we noticed that differences in branching number between two adjacent brightness points below 220 tended to increase within this patient sample.

The brightness threshold of 220 was empirically established during preliminary testing on a subset of representative images, with the aim of identifying the lowest value that reliably captured tear-ferning branch structures while minimizing background noise. After selection, this threshold was applied uniformly across all images and was not adjusted on an individual basis.

**Figure 4:**
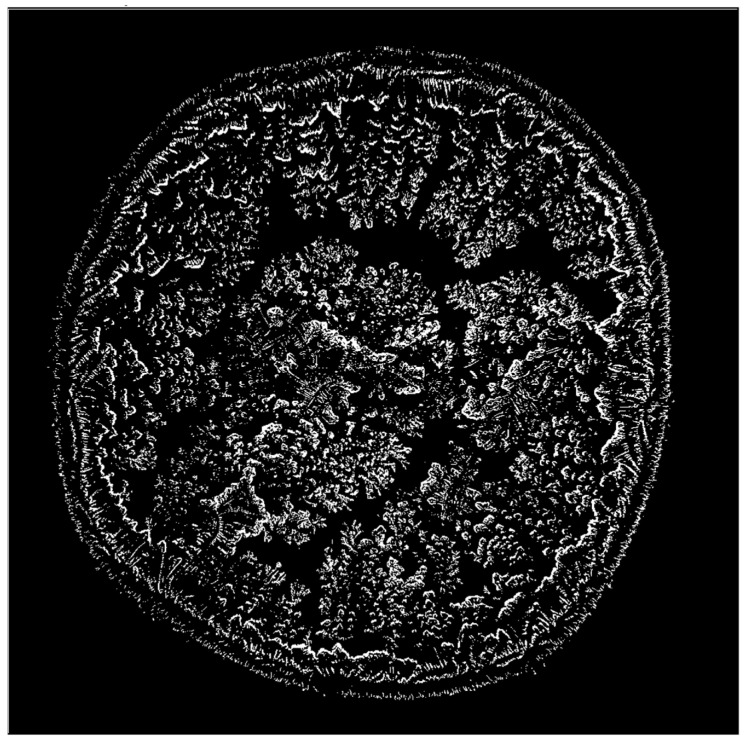
Image converted into a binary format for structural analysis.

**Figure 5:**
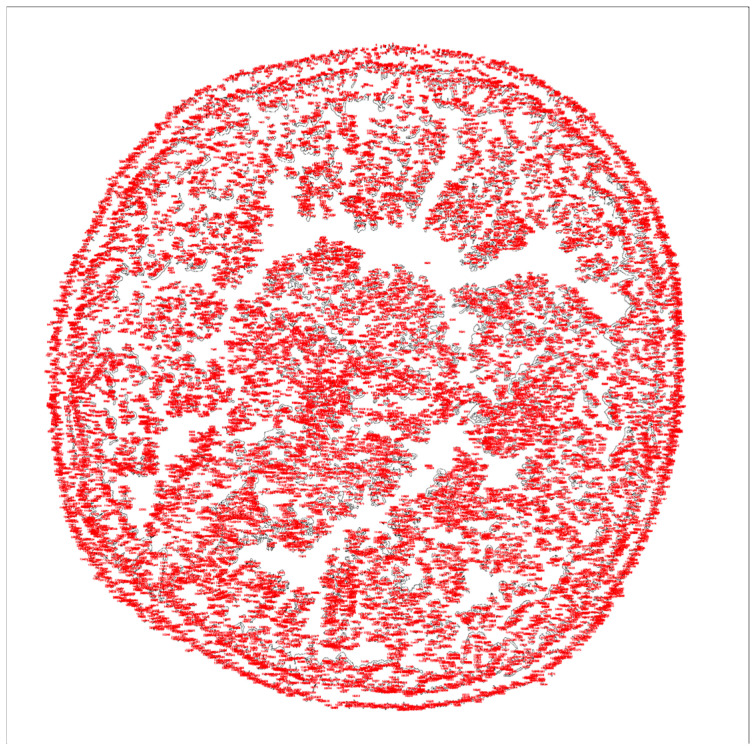
Quantification of branching structures using ImageJ2.

All images were subsequently processed in ImageJ2 using a successive workflow that included color threshold adjustment, binary transformation, and particle analysis (Figure 4 and Figure 5).

A total of 61 patients were included in the study, ranging in age from 25 to 72 years (median [interquartile range] = 39.0 [26] years), with age values showing a non-normal distribution (Shapiro–Wilk *p* < 0.001). These data indicate that the sample consisted primarily of young adults.

As shown in Table 1, both age and BMI increase progressively across the Masmali severity classes. Because age is a well-established factor influencing tear film quality, this pattern suggests a potential confounding effect, which we further examined through age-adjusted analyses.

After adjusting for age, the association between BMI and Masmali classification diminished and was no longer statistically significant. This indicates that the relationship seen in the unadjusted analyses may be partly explained by age-related changes in the tear film rather than by BMI alone (Table 2).

Statistical analyses consistently revealed significant differences among the Masmali classes in both BMI and tear-ferning branch counts. Full statistical details are presented in the Supplementary Materials.

The Kruskal–Wallis test was used to examine the association between Masmali classes and BMI. The analysis revealed statistically significant differences between at least two Masmali classes (*p* = 0.024).

Post hoc Dunn tests showed that patients in Masmali class 2 had a significantly higher BMI compared to those in class 0 (*p* = 0.009), while no statistically significant differences were identified between classes 0–1 or 1–2.

A One-way ANOVA was used to assess differences in tear brightness 220 branching counts across Masmali classes. The analysis showed a statistically significant difference between Masmali classes 0, 1, and 2 in the number of branches identified in dried tear samples at a brightness threshold of 220. Accordingly, as the Masmali score increases, the number of branches decreases significantly (Table 3).

The mean branch count decreases from 13,842 in Masmali class 0 to 9296 in class 2, illustrating the loss of the crystallization pattern with advancing tear film dysfunction (Table 4).

**Figure 6:**
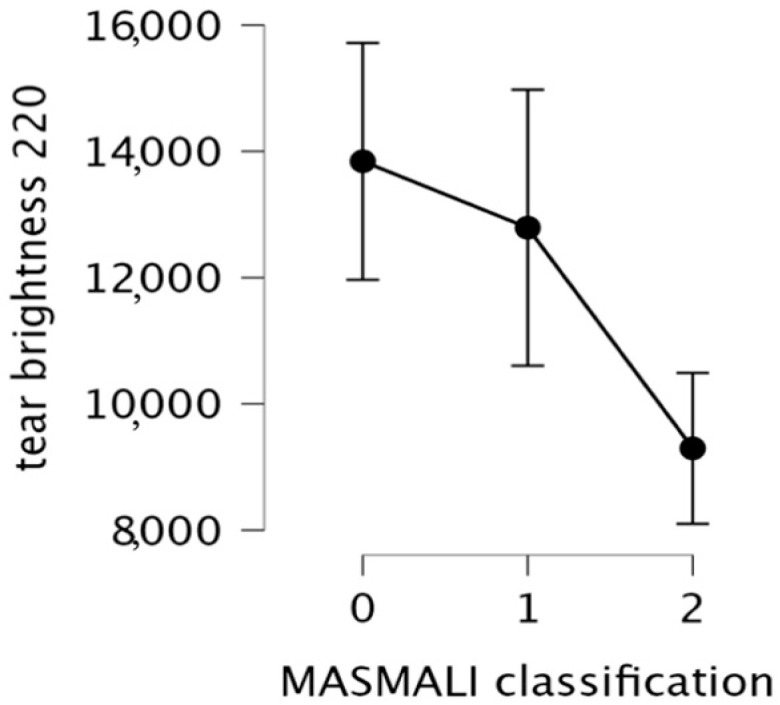
Descriptives plots.

The descriptive plot illustrates an inverse relationship between the Masmali score and the number of tear-fern branches at Brightness 220. Thus, as the tear film becomes increasingly compromised, the ferning patterns exhibit progressive simplification, characterized by a marked reduction in branching (Figure 6).

Post hoc tests indicated no significant difference between classes 0 and 1 (*p* = 0.682), but significant reductions in branch number were found in comparisons with class 2, supporting the conclusion that severe tear film dysfunction is associated with a substantial loss of branching.

## 4. Discussion

Obesity represents one of the diagnostic criteria of metabolic syndrome. This supports the hypothesis of a correlation between increased body weight and dry eye disease. In this regard, the study by Erdur et al., “The relationship between metabolic syndrome, its components, and dry eye: a cross-sectional study”, showed a positive correlation between higher Ocular Surface Disease Index (OSDI) scores, increased tear film osmolarity, and the presence of metabolic syndrome. The Tear Ferning Test (TFT) represents a cost-effective alternative to measuring tear osmolarity [10].

This pathophysiological relationship is further supported by the study “A Possible Association Between Dry Eye Symptoms and Body Fat: A Prospective, Cross-Sectional Preliminary Study”. In that investigation, Ho et al. demonstrated a positive correlation between body fat percentage and dry eye symptoms in a group of 305 patients [11].

A study conducted by Alanazi et al. reported that although the quantity of tear film distribution in patients with obesity is comparable to that of normal-weight subjects, the quality of the tear film is significantly altered in those with increased body weight [12].

On the other hand, Masmali et al. concluded that crystallization patterns identified in the tear ferning test have been used to assess tear film quality in patients with uncontrolled diabetes [13].

Moreover, the number of branches identified in dried tear ferning patterns appears to correlate with sarcoidosis-induced dry eye, as evaluated by Sandu et al. In this study, the branching count quantified using ImageJ2 was significantly higher in patients with sarcoidosis-associated dry eye compared with those with idiopathic dry eye and with the control group [14].

Essam et al. showed that the crystallization patterns identified in the Tear Ferning Test represent a sensitive parameter for the qualitative assessment of tear film composition, as they are influenced by the chemical makeup of the tear fluid [15].

A recent study reported the absence of any diurnal influence on tear crystallization patterns. Nevertheless, because patient weighing was required in our protocol, we elected to assess patients in a fasting state, without prior ingestion of water or food, to ensure greater accuracy of anthropometric measurements [16]. Not only patient weighing, but also other physiological parameters may be altered by the intake of fluids or food. Both peppermint tea and green tea were shown to exert a detrimental effect on tear film stability in normoweight patients [17,18].

BMI is a well-established measure and is routinely used in clinical practice to evaluate weight status. However, more advanced methods—such as bioimpedance analysis and digital anthropometry—offer greater sensitivity for detecting variations in adipose tissue distribution [19].

The findings of this study are consistent with those reported in the existing literature. Alanazi et al. showed a positive correlation between increasing BMI and tear film impairment, as quantified by the Tear Ferning Test (TFT) [20].

High BMI values correlated with more advanced tear-ferning classes and a marked decrease in the number of fern-like branches. These results align with prior research showing that excess body weight can impair the tear film, indicating that systemic metabolic status may also manifest in tear crystallization patterns [21,22,23].

Beyond its systemic associations, dry eye syndrome is known to significantly impair daily functioning and overall quality of life, which underlines the importance of detecting early ocular surface changes even in systemic conditions such as obesity [24].

Previous research has likewise shown that ocular surface parameters can improve following bariatric surgery [25]. However, such findings should be interpreted cautiously, as a known postoperative complication is vitamin A deficiency secondary to malabsorption, which may clinically manifest as dry eye due to tear film compromise [26]. Prophylactic oral vitamin A administration improves tear quality, although it does not enhance tear volume [27].

The originality of this study stems from its objective, quantitative assessment of tear-ferning morphology, rather than from the BMI–tear film association itself, which has already been documented. Through the use of standardized digital image processing in ImageJ2, we quantified the number of ferning branches and linked these values to clinically graded tear film severity. This quantitative method minimizes the subjectivity of traditional visual grading systems and offers a reproducible framework for examining microstructural changes in the tear film.

Within this context, the correlations observed between BMI and tear-ferning results should be viewed as supportive evidence rather than definitive proof. Our findings extend previous work by showing that quantitative ferning metrics capture the severity of tear film dysfunction and may also reflect systemic influences such as elevated body mass. Tear film DPT holds considerable potential as a cost-effective diagnostic method for systemic pathologies. Future studies are required to assess the correlations between variations in branching patterns across different biofluids collected from the same patient.

Using a fixed brightness threshold can introduce some variability into image-based analyses. Even so, applying the same threshold to all samples improves reproducibility and limits operator-dependent bias. Future research could investigate automated or adaptive thresholding methods to enhance generalizability under varying imaging conditions. Although efforts were made to enhance the reproducibility standards of the test, the tear film remains subject to a wide range of multimodal influences. Emotional states, meteorological conditions, and physiological hormonal fluctuations may each induce measurable alterations in both the quality and quantity of tears. Furthermore, a larger sample size is required to allow for broader extrapolation of the findings.

This study shows an association between BMI and tear-ferning alterations in the unadjusted analyses, while also underscoring the role of age as a key confounding factor. Because of the cross-sectional design, these results should be understood as associative rather than causal. Lifestyle factors are likewise reflected in the tear composition of study participants. Thus, imposing a controlled dietary regimen prior to tear sample collection may be necessary to increase the methodological rigor of the research. Further research is required to clarify the links between variations in biological fluid composition and weight status.

## 5. Conclusions

The Tear Ferning Test (TFT) may be a useful clinical research tool for exploring how excess body weight relates to tear film alterations. Systemic inflammation, which frequently accompanies higher body weight, has been proposed as a possible contributor to ocular surface changes. Nevertheless, because this study is cross-sectional, the results should be viewed as associative rather than causal. Additional longitudinal research with comprehensive assessments is needed to clarify the underlying mechanisms.

## Figures and Tables

**Table 1:** Descriptive statistics for Age and BMI according to Masmali classification.

Variable	Class 0 (n = 15)	Class 1 (n = 16)	Class 2 (n = 30)
Age (years) *	29.0 (14.5)	43.0 (27.8)	43.0 (23.5)
BMI (kg/m^2^) *	22.8 (7.8)	29.2 (9.0)	32.5 (11.6)

* Data are expressed as median (interquartile range, IQR). Normality was assessed with the Shapiro–Wilk test and both variables showed non-normal distribution (*p* < 0.05).

**Table 2:** Statistical correlation between BMI-MASMALI classification.

	Pearson		Spearman	
	r	*p*	rho	*p*
**BMI-MASMALI classification**	0.244	0.060	0.239	0.066

Conditioned on variables: Age.

**Table 3:** One-way ANOVA between tear brightness 220 branching counts and Masmali classification *.

							95% CI for η^2^	
Cases	Sum of Squares *	df	Mean Square	F	*p*	η^2^	Lower	Upper
MASMALI classification	2.528 × 10^+8^	5	1.264 × 10^+8^	10.331	<0.001	0.263	0.080	0.426
Residuals	7.097 × 10^+8^	58	1.224 × 10^+7^					

* Type III Sum of Squares.

**Table 4:** Descriptive statistics for tear 220 brightness.

MASMALI Classification	N	Mean	SD	SE	Coefficient of Variation
0	15	13,842.000	3385.716	874.188	0.245
1	16	12,789.750	4099.864	1024.966	0.321
2	30	9296.367	3200.542	584.336	0.344

## Data Availability

The original contributions presented in this study are included in the article. Further inquiries can be directed to the corresponding author.

## Supplementary Materials

The following supporting information can be downloaded at: https://www.mdpi.com/article/10.3390/life16020210/s1.

## Abbreviations

The following abbreviations are used in this manuscript:

ANOVA	Analysis of variance
BMI	Body mass index
DPT	Diagnostic patterning test
OSDI	Ocular surface disease index
IQR	Interquartile range
TFT	Tear ferning test
