# Supplementary material for: Alterations in the Crystallization Pattern of Tear Fluid Induced by Increases in the Body Mass Index
Source: Life (Basel). 2026 Jan 27;16(2):210. doi: 10.3390/life16020210 (PMC12942425; doi:10.3390/life16020210)
Supplement: Supplementary file 1 [file life-16-00210-s001.zip › life-4076493-supplementary.pdf]

ANOVA - BMI

								95% CI for η^2	
Homogeneity Correction	Cases	Sum of Squares*	df	Mean Square	F	p	η^2	Lower	Upper
None	MASMALI classification	376.460	2.000	188.230	3.854	0.027	0.117	0.000	0.272
	Residuals	2833.090	58.000	48.846					
Brown-Forsythe	MASMALI classification	376.460	2.000	188.230	3.934	0.026	0.117	0.000	0.272
	Residuals	2833.090	46.900	60.407					
Welch	MASMALI classification	376.460	2.000	188.230	3.617	0.038	0.117	0.000	0.272
	Residuals	2833.090	31.602	89.648					

*Type III Sum of Squares

Test for Equality of Variances (Levene's)

F	df1	df2	p
0.402	2.000	58.000	0.671

Kruskal-Wallis Test

					95% CI for Rank η^2	
Factor	Statistic	df	p	Rank η^2	Lower	Upper
MASMALI classification	7.448	2	0.024	0.094	0.000	0.362

Dunn's Post Hoc Comparisons - MASMALI classification*

Comparison	z	Wi	Wj	rrb	p	pbonf	pholm
0 - 1	-0.944	22.167	28.188	0.208	0.345	1.000	0.345
0 - 2	-2.628	22.167	36.917	0.478	0.009	0.026	0.026
1 - 2	-1.588	28.188	36.917	0.292	0.112	0.337	0.224

Standard (HSD). Bootstrapped Post Hoc Comparisons - MASMALI classification

			**95% bca† CI**						
		**Mean Difference**	**Lower**	**Upper**	**SE**	**df**	**bias**	**t**	**p_{tukey}**
0	1	1009.092	-1550.934	3686.516	1325.361	58	-54.349	0.837	0.682
	2	4449.819	2625.072	6851.757	1057.607	58	-35.468	4.109	< .001***
1	2	3519.710	1051.128	5836.095	1152.185	58	18.881	3.226	0.006 **

** p < .01, *** p < .001

† Bias corrected accelerated.

Note. Bootstrapping based on 1000 successful replicates.

Note. Mean Difference estimate is based on the median of the bootstrap distribution.

Note. P-value adjusted for comparing a family of 3 estimates.
